# Efficacy of combined modality conversion therapy for HCC with portal vein tumor thrombus: a retrospective cohort study

**DOI:** 10.3389/fimmu.2026.1749090

**Published:** 2026-06-18

**Authors:** Jian Huang, Fu-Chen Liu, Li Li, Wei Dong, Sheng-Xian Yuan, Fang-Ming Gu, Bei-Ge Jiang, Ze-Ya Pan

**Affiliations:** 1 The Third Department of Hepatic Surgery, Eastern Hepatobiliary Surgery Hospital, Naval Medical University, Shanghai, China; 2 Department of Nephrology, Eastern Hepatobiliary Surgery Hospital, Naval Medical University, Shanghai, China

**Keywords:** conversion therapy, hepatocellular carcinoma, portal vein tumor thrombus, salvage hepatectomy, survival

## Abstract

**Background:**

Unresectable hepatocellular carcinoma (uHCC) patients with portal vein tumor thrombus (PVTT) have a substandard prognosis. This study elucidated whether conversion therapy can alleviate unresectable tumors and increase the rate of salvage hepatectomy, as well as identify predictors of better prognosis.

**Methods:**

This retrospective study analyzed 159 patients with uHCC and PVTT who underwent conversion therapy, predominantly combining systemic treatments with transarterial chemoembolization (TACE). Primary endpoints were progression-free survival (PFS) and overall survival (OS). Secondary endpoints included tumor response, surgical conversion rate, and outcomes in the surgical cohort.

**Results:**

The data showed that the objective response rates (ORR) for the entire cohort (n = 159) were 42.1%, while the disease control rate was 62.3%. The median PFS and OS were respectively determined to be 9.6 and 17.7 months. Moreover, 40 patients (25.2%) were successfully converted and underwent salvage hepatectomy. In addition, the surgical subgroup indicated a markedly improved median recurrence-free survival (RFS; 10.1 months) and OS (30 months). Multivariate analysis revealed that salvage hepatectomy was an independent protective factor for both PFS (95% CI: 0.341 - 0.845, HR = 0.537, *p = 0.007*) and OS (95% CI: 0.273 - 0.777, HR = 0.460, *p = 0.004*). Whereas for poorer outcomes, an independent predictor was advanced Cheng’s PVTT classification (OS: HR = 1.719, *p < 0.001*). Multivariate logistic regression showed that a single tumor (OR = 0.402, *p = 0.024*) and a lower baseline aspartate aminotransferase level (OR = 0.453, *p = 0.038*) were independent predictors of successful conversion to surgery. Of the 40 surgical patients, 11 (27.5%) attained a pathologic complete response (pCR). Although patients with pCR showed a numerically favorable survival trend (median RFS of 14.0 months vs. 4.5 months for major pathological response), this difference did not reach statistical significance. Adverse events associated with treatment were manageable, with grade ≥ 3 adverse events observed in 19.5% of patients.

**Conclusion:**

This retrospective study evaluated the combined effect of systemic therapy and TACE as an efficient conversion therapy for uHCC patients with PVTT. The findings revealed a substantial response rate, facilitating curative-intent surgery in a substantial percentage of patients. Salvage hepatectomy was associated with improved survival in selected patients who responded to conversion therapy.

## Introduction

Hepatocellular carcinoma (HCC) is the 3^rd^ major cause of death by cancer globally. Furthermore, its incidence is specifically high in East Asia because of the prevalence of chronic hepatitis B virus (HBV) infection (1). About 10–40% patients indicate portal vein tumor thrombus (PVTT) at diagnosis, which is a hallmark of advanced disease and a critical negative prognostic factor. Moreover, if left untreated, PVTT has been linked with only 2.7 months of median survival (2). The Barcelona Clinic Liver Cancer (BCLC) staging system categorizes PVTT patients as advanced-stage (BCLC-C). These patients have traditionally been treated with systemic therapy (3).

The treatment approach for advanced HCC has been improved by the development of immune checkpoint inhibitors (ICIs) and targeted treatments. For instance, the combination of atezolizumab and bevacizumab markedly elevates overall survival (OS), establishing a novel first-line standard of therapy (4). Furthermore, various combinations of TKIs and ICIs have indicated significant efficacy, expanding the therapeutic potential (5, 6). Despite these advances, the prognosis of PVTT patients remains substandard, and the primary treatment involves systemic therapy. Transarterial chemoembolization (TACE) is an efficacious locoregional treatment that promotes ischemic necrosis in intrahepatic neoplasms. Multiple studies have demonstrated that co-treatment with TACE and conventional systemic treatments can increase objective response rates (ORR) and facilitate successful surgical conversion in patients with advanced HCC (7, 8).

However, several critical questions remain unanswered regarding the purported efficacy of conversion therapy. The optimal integration of systemic and locoregional therapy is still uncertain, and reliable predictors for successful surgical conversion warrant further research. Furthermore, the predictive importance of the pathological response observed in resected tissues following neoadjuvant treatment, including pathological complete response (pCR), requires additional validation in HCC patients with PVTT.

Therefore, this retrospective investigation studied the effectiveness and safety of a combined-modality conversion strategy, primarily integrating TACE with different systemic therapies, in unresectable HCC (uHCC) patients with PVTT. Furthermore, the rate of successful conversion to salvage hepatectomy was assessed, successful conversion-predicting factors were identified, and the survival outcomes of the entire cohort were measured, specifically in patients who underwent surgery to elucidate their survival benefits.

## Materials and methods

### Study subjects

This retrospective study evaluated uHCC patients treated at the Eastern Hepatobiliary Surgery Hospital (EHBH) between January 2021 and October 2023. The patient’s consent was acquired before the study, and their clinical and pathological data were prospectively maintained in a database. This study followed the Helsinki Declaration and was authorized by the Ethical Board of EHBH.

The uHCC patients (1) who were diagnosed with PVTT *via* radiography or histopathology (2), with an Eastern Cooperative Oncology Group (ECOG) performance status (PS) of 0 or 1 (3), with at least one measurable lesion based on the modified Response Evaluation Criteria in Solid Tumors (mRECIST), and (4) with Child-Pugh class A liver function were included in the study. Whereas individuals with (1) an ECOG PS score > 1 (2), concurrent malignancies (3), extrahepatic metastases (4), metastasis of major vascular structures (*e.g.*, inferior vena cava, hepatic vein), or presence of macroscopic bile duct tumor thrombus, and (5) incomplete clinical data were not included in the study. A multidisciplinary team (MDT) led by surgeons determined treatment allocation. Decisions were made according to recognized clinical criteria, incorporating an extensive evaluation of the patient's overall state, tumor load, laboratory data, and imaging findings.

### Data collection

Each patient’s baseline clinical characteristics were systematically assessed before the treatment. These data encompassed demographic information (sex, age), hepatitis virus status, medical history (such as liver cirrhosis), liver function status (evaluated by albumin-bilirubin [ALBI] levels and other liver function tests), and circulating tumor biomarker levels. The ALBI score was employed to evaluate liver functional reserve for the entire cohort. Compared to the Child-Pugh score, ALBI offers a more objective and standardized assessment, particularly in patients without overt cirrhosis but with chronic viral hepatitis, as it eliminates the subjectivity associated with assessing ascites and encephalopathy. The following formula was employed to measure the ALBI score: (log_10_ bilirubin [μmol/L] × 0.66) + (albumin [g/L] × -0.085) (9). Furthermore, PVTT diagnosis was confirmed *via* characteristic radiological features on preoperative imaging or by intraoperative and postoperative histopathological examination. Then, based on Cheng's classification system, PVTT was categorized (10, 11). This system is significantly employed in China and stratifies PVTT into four distinct types depending on the anatomical depth of the tumor thrombus present in the portal venous system. Type I includes segmental or more distant portal vein branches; Type II comprises the right or left portal vein; Type III indicates invasion of the primary portal vein trunk; and Type IV implies thrombus extension into the superior mesenteric vein.

### Treatment regimens for conversion therapy

The conversion therapy integrated systemic and locoregional approaches. Systemic treatments included targeted therapy and immunotherapy. Targeted agents comprised oral tyrosine kinase inhibitors sorafenib (400 mg twice daily), lenvatinib (dosed at 8 or 12 mg once daily based on bodyweight), apatinib (250 mg once daily), and donafenib (200 mg twice daily), and intravenous bevacizumab (15 mg/kg every 3 weeks). Immunotherapies were administered every 3 weeks, including intravenous checkpoint inhibitors like sintilimab (200 mg), tislelizumab (200 mg), pembrolizumab (200 mg), atezolizumab (1200 mg), camrelizumab (200 mg), and toripalimab (240 mg). The selection of systemic agents was individualized by the multidisciplinary team according to the drug accessibility, patient tolerance, liver function, tumor burden, and clinician judgment in a real-world clinical setting.

The primary locoregional treatment was TACE, which was carried out *via* the Seldinger technique, using percutaneous access to the femoral artery for the selective catheterization of tumor-feeding arteries. A combination of chemotherapeutic medicines (epirubicin, fluorouracil, and platinum) and embolic agents (lipiodol and gelatin sponge) was subsequently administered. The chemotherapy dose was adjusted for each patient per tumor status, overall health state, and body surface area. To ensure the safety of TACE in patients with PVTT (especially Cheng’s types II and III), we employed super-selective catheterization using microcatheters to target the tumor-feeding branches while sparing the non-tumorous liver parenchyma. Additionally, TACE was only performed in patients with adequate collateral circulation around the obstructed portal vein (cavernous transformation) or those with well-compensated liver function (Child-Pugh Class A). For patients with main portal vein obstruction, a reduced dose of embolic agents and a more personalized injection speed were applied to prevent extensive liver necrosis. The initial TACE procedure was conducted 2 to 4 weeks after starting systemic medication. Further TACE sessions were conducted every 6 to 8 weeks, based on radiological evaluation. Treatment continued until TACE became unfeasible or the patient encountered intolerable toxicity.

Given the real-world retrospective nature of this study and the fragmentation of systemic regimens across multiple agents and combinations, the analysis was designed to evaluate the overall effectiveness of the combined conversion strategy rather than to compare the efficacy of individual systemic regimens.

### Salvage hepatectomy

Patients who attained a complete response (CR) or partial response (PR) according to mRECIST were assessed for salvage hepatectomy. MDT reached the final decision following a thorough evaluation of the expected surgical complexity, degree of resection, and operative risks.

Resectability conversion was determined by the satisfaction of all the following requirements: (1) an ECOG PS score of 0 or 1; (2) complete necrosis of tumor thrombus in the primary portal vein along with it associated branches; (3) potential for complete (R0) resection (12) with adequate future liver remnant (FLR) volume; (4) Child-Pugh class A hepatic function; (5) lack of extrahepatic metastases; and (6) absence of any surgical contraindications.

Before the scheduled hepatectomy, systemic therapies were stopped for a specified duration. Tyrosine kinase inhibitors (*e.g.*, Lenvatinib, sorafenib) were discontinued for a minimum of 1 week, anti-PD-1 antibodies for at least 4 weeks, and bevacizumab for at least 6 weeks. In the assessment of pathological response, pCR was characterized by the total absence of viable tumor cells in the excised tumor material, while major pathological response (MPR) was defined as ≤ 10% (13).

### Endpoints

The study’s primary endpoints were OS and progression-free survival (PFS), where the latter was described as the period from the treatment start to the initial documentation of disease progression, evaluated following the mRECIST or mortality from any cause, whichever transpires first. The OS was defined as the time elapsed between the initiation of treatment and death from any cause. Secondary endpoints encompassed tumor response and, for the surgical cohort, recurrence-free survival (RFS). The assessment of tumor response was carried out using the ORR and the disease control rate (DCR). RFS was described as the duration between the date of surgery and the first confirmed instance of tumor recurrence, while the adverse events (AEs) were confirmed by using Common Terminology Criteria for Adverse Events (CTCAE) version 5.0. In this retrospective study, AEs were recorded as treatment-related toxicities occurring during the entire course of conversion therapy. Because systemic therapy and TACE were administered in combination and some toxicities overlapped, individual events could not always be definitively attributed to a single modality.

### Follow-up

Contrast-enhanced computed tomography (CT) or magnetic resonance imaging (MRI) was employed to evaluate tumor response every 6 to 8 weeks. Furthermore, additional imaging was carried out based on the symptoms or signs indicating disease progression. The mRECIST was adopted for efficacy assessment, and responses were categorized as CR, stable disease (SD), PR, or progressive disease (PD). During each follow-up visit, laboratory evaluations were performed, encompassing urinalysis, serum chemistry panels (liver and renal function), complete blood counts, thyroid function assessments, cardiac enzyme analysis, and HBV-DNA quantification.

Moreover, serum tumor markers, particularly alpha-fetoprotein (AFP) and Prothrombin Induced by Vitamin K Absence or Antagonist-II (PIVKA-II), were also observed. The final follow-up date for all patients was September 2024.

### Statistical measurements

Categorical variables were depicted as percentages and frequencies, whereas range and median were used for continuous variables. Comparisons of categorical variable differences were conducted using the chi-squared (χ^2^) test or Fisher’s exact test, as appropriate. The Kaplan-Meier method was employed for survival curves generation, which were analyzed using the log-rank test. Univariate and multivariate Cox proportional hazards models were utilized to discover prognostic markers for survival. For the multivariate analysis, a rigorous variable selection strategy was applied. Variables demonstrating statistical significance (*p*  < 0.05) in the univariate analysis were included in the multivariate Cox regression model. To avoid multicollinearity, inherently linked variables were evaluated, and only the most strongly associated parameter from the univariate analysis was entered into the respective multivariate model. Variables that lost statistical significance (*p* > 0.05) after covariate adjustment were considered non-independent factors. For clarity and conciseness, only variables that remained independent predictors are presented in the final multivariate results. In addition, a multivariate logistic regression assay was conducted to assess independent predictors for successful conversion to surgery. To address potential immortal time bias, a time-dependent Cox proportional hazards regression model was performed. In this model, salvage hepatectomy was incorporated as a time-dependent covariate to provide a more accurate assessment of its impact on OS and PFS. The proportional hazards (PH) assumption for the Cox regression models of both OS and PFS was assessed using Schoenfeld residual tests. In addition, receiver operating characteristic (ROC) curve analysis was performed to evaluate the discriminative ability of the multivariable logistic regression model for successful conversion to salvage hepatectomy, and the area under the curve (AUC) with 95% confidence interval (CI) was calculated. All statistical assays were two-sided, and a *p-value < 0.05* reflected statistical significance. Figures were generated *via* GraphPad Prism version 9.0 (San Diego, CA, USA), and statistical measurements were carried out with SPSS version 26.0 (IBM Corp., Armonk, NY, USA).

## Results

### Baseline characteristics of uHCC patients with PVTT

In total, 159 patients with uHCC and PVTT were analyzed in this investigation, with **Table 1** showing the baseline features of the cohort. In the whole cohort, 93.1% patients were male (n = 148), with 51.4 ± 11.1 years of mean age. Furthermore, 137 patients (86.2%) were positive for Hepatitis B surface antigen (HBsAg), with 51 (32.1%) exhibiting HBV-DNA levels > 2000 IU/mL. Moreover, liver cirrhosis was observed in 84 patients (52.8%). The majority of patients exhibited satisfactory liver function, as evidenced by Albumin-Bilirubin (ALBI) grade 1 (n = 64, 40.3%) or grade 2 (n = 87, 54.7%), with just 8 patients (5.0%) categorized as ALBI grade 3. The tumor marker assessments showed that AFP levels ≥ 400 ng/mL in 90 patients (56.6%), AFP-L3 was positive in 77 (48.4%) patients, and PIVKA-II levels ≥ 400 mAU/mL in 102 (64.2%) patients. It was also observed that 61 patients (38.4%) had a maximum tumor diameter > 10 cm, while 68 (42.8%) patients had multiple tumors. Cheng's classification indicated that there were 49 (30.8%) patients with type I PVTT, 71 (44.7%) with type II, 37 (23.3%) with type III, and 2 (1.3%) with type IV. The primary locoregional intervention was TACE, administered in 146 individuals (91.8%). Further, 40 patients (25.2%) were efficiently down-staged by conversion therapy and later received curative salvage liver resection.

**Table 1:** Clinical characteristics of unresectable HCC patients with PVTT.

Variables	Total patients	Non conversion	Conversion	P value
	n=159	n=119	n=40	
Sex					
Male	148 (93.1%)	111 (93.3%)	37 (92.5%)	1.000
Female	11 (6.9%)	8 (6.7%)	3 (7.5%)	
Age, y					
≤60	122 (76.7%)	90 (75.6%)	32 (80.0%)	0.727
>60	37 (23.3%)	29 (24.4%)	8 (20.0%)	
HBsAg					
Negative	22 (13.8%)	13 (10.9%)	9 (22.5%)	0.116
Positive	137 (86.2%)	106 (89.1%)	31 (77.5%)	
HBV-DNA load, IU/mL					
<2000	108 (67.9%)	78 (65.5%)	30 (75.0%)	0.362
≥2000	51 (32.1%)	41 (34.5%)	10 (25.0%)	
ALBI grade					
1	64 (40.3%)	43 (36.1%)	21 (52.5%)	0.075
2	87 (54.7%)	68 ( 57.1%)	19 (47.5%)	
3	8 (5.0%)	8 (6.7%)	0 (0.0%)	
TB, μmol/L					
<34.2	147 (92.5%)	107 (89.9%)	40 (100.0%)	0.081
≥34.2	12 (7.5%)	12 (10.1%)	0 (0.0%)	
AST, IU/L					
≤40	77 (48.4%)	52 (43.7%)	25 (62.5%)	0.061
>40	82 (51.6%)	67 (56.3%)	15 (37.5%)	
ALT, IU/L					
≤40	82 (51.6%)	61 (51.3%)	21 (52.5%)	1.000
>40	77 (48.4%)	58 (48.7%)	19 (47.5%)	
ALB, g/L					
≥40	75 (47.2%)	51 (42.9%)	24 (60.0%)	0.090
<40	84 (52.8%)	68 (57.1%)	16 (40.0%)	
AFP-L3					
<10	92 (57.9%)	70 (58.8%)	22 (55.0%)	0.811
≥10	67 (42.1%)	49 (41.2%)	18 (45.0%)	
AFP, μg/L					
<400	69 (43.4%)	50 (42.0%)	19 (47.5%)	0.674
≥400	90 (56.6%)	69 (58.0%)	21 (52.5%)	
PVK-II, mAU/mL					
<400	57 (35.8%)	39 (32.8%)	18 (45.0%)	0.228
≥400	102 (64.2%)	80 (67.2%)	22 (55.0%)	
Tumor size, cm					
<10	98 (61.6%)	78 (65.5%)	20 (50.0%)	0.118
≥10	61 (38.4%)	41 (34.5%)	20 (50.0%)	
Tumor number					
Single	91 (57.2%)	62 (52.1%)	29 (72.5%)	***0.038***
Multiple	68 (42.8%)	57 (47.9%)	11 (27.5%)	
TACE					
No	13 (8.2%)	10 (8.4%)	3 (7.5%)	1.000
Yes	146 (91.8%)	109 (91.6%)	37 (92.5%)	
Liver cirrhosis					
No	75 (47.2%)	53 (44.5%)	22 (55.0%)	0.335
Yes	84 (52.8%)	66 (55.5%)	18 (45.0%)	
Cheng's PVTT type					
I	49 (30.8%)	35 (29.4%)	14 (35.0%)	0.748
II	71 (44.7%)	53 (44.5%)	18 (45.0%)	
III	37 (23.3%)	29 (24.4%)	8 (20.0%)	
IV	2 (1.3%)	2 (1.7%)	0 (0.0%)	

HBsAg, hepatitis B surface antigen;TB, total bilirubin; ALB, albumin; ALT, alanine transaminase; AST, aspartate aminotransferase; HBV-DNA, hepatitis B virus deoxyribonucleic acid; ALBI, Albumin-bilirubin grade; AFP, a-fetoprotein; AFP-L3, LCA-reactive alpha-fetoprotein isoform; MVI, microvascular invasion; PVTT, portal vein tumor thrombus; PIVKA-II, Protein Induced by Vitamin K Absence or Antagonist-II; TACE, transarterial chemoembolization P<0.05 was defined as statistical significance and indicated in bold italics.

### Treatment efficacy and survival outcomes

**Figure 1:**
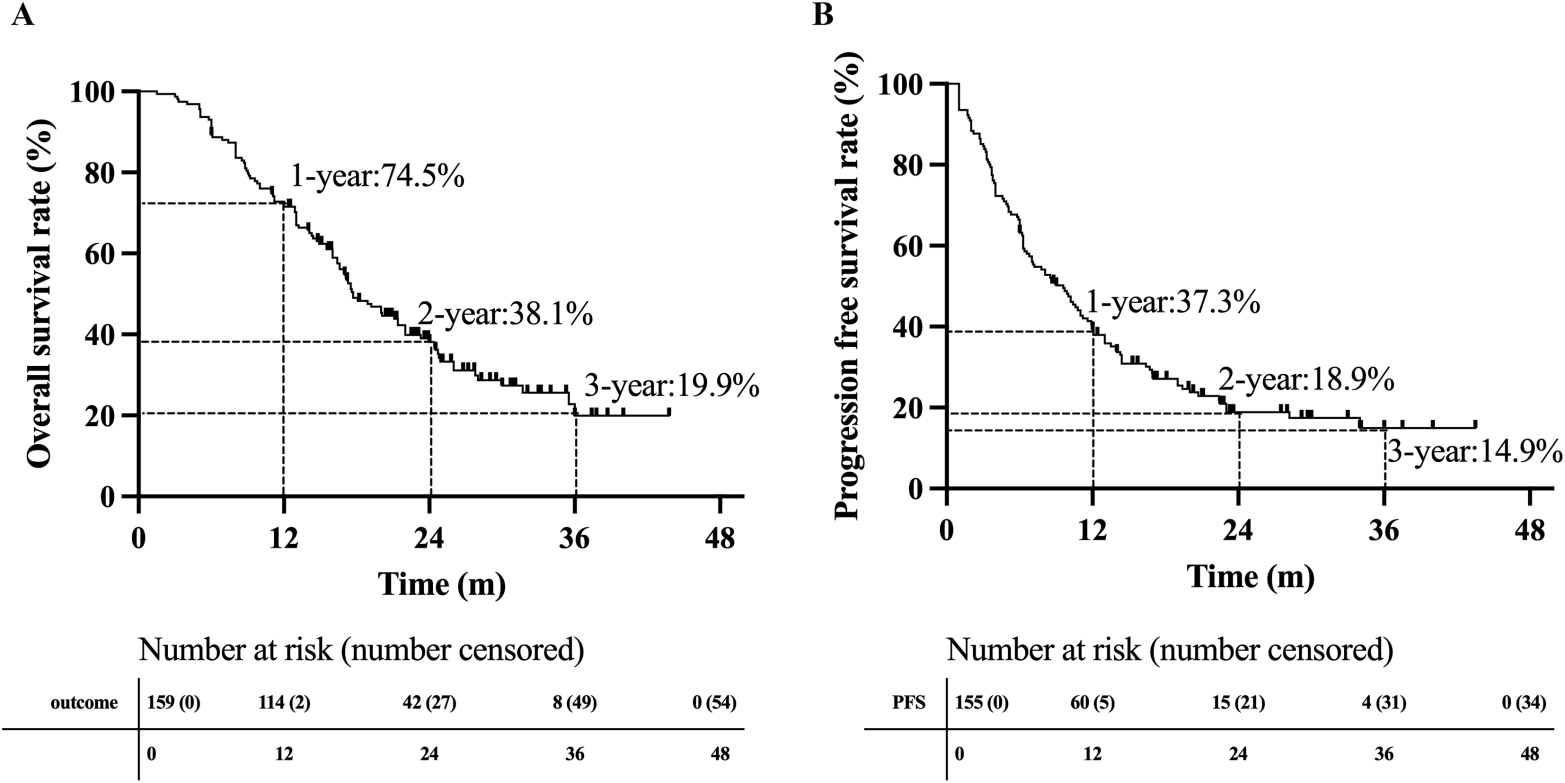
Kaplan-Meier curves for all uHCC patients with Portal Vein Tumor Thrombus. **(A)** Overall survival (OS). **(B)** Progression free survival (PFS). HCC, hepatocellular carcinoma.

**Table 2** summarizes the best objective responses to treatment for the entire cohort. The data showed that CR, PR, SD, and PD were attained in 14 (8.8%), 53 (33.3%), 32 (20.1%), and 60 (37.7%) patients. This yielded an ORR of 42.1% (n = 67) and a DCR of 62.3% (n = 99) for the whole cohort. In the 40 patients who underwent salvage liver resection, 11 (27.5%) achieved a CR, whereas 29 (72.5%) achieved a PR. Among the 60 non-surgical patients who progressed, 26 cases (43.3%) had intrahepatic parenchymal progression, 15 cases (25%) had PVTT extension, 9 cases (15%) had simultaneous progression of both parenchymal tumors and PVTT, and 10 cases (16.7%) developed new extrahepatic metastases. All the patients were monitored for 2 to 45 months (median 31 months). The cumulative OS rates of the whole cohort at 1, 2, and 3 years were 74.5%, 38.1%, and 19.9%, respectively, while PFS rates were 37.3%, 18.9%, and 14.9%, respectively (**Figures 1A, B**). The median PFS and OS were 9.6 and 17.7 months, respectively.

**Table 2:** Tumor response in all patients.

Tumor response	Total	Conversion	Non-conversion
	n=159	n=40	n=119
CR	14 (8.8%)	11 (27.5%)	3 (2.5%)
PR	53 (33.3%)	29 (72.5%)	24 (20.2%)
SD	32 (20.1%)	N/A	32 (26.9%)
PD	60 (37.7%)	N/A	60 (50.4%)
ORR	67 (42.1%)	N/A	27 (22.7%)
DCR	99 (62.3%)	N/A	59 (49.6%)
Median OS (m)	17.7	30	16.4
Median PFS (m)	9.6	13.5	7

CR, complete response; PR, partial response; SD, stable disease; PD, progressive disease; ORR, objective response rate; DCR, disease control rate; OS, overall survival; PFS, progression-free survival N/A: Not Applicable.

### Independent risk factors of PFS and OS in the entire cohort

The univariate and multivariate analyses results are detailed in **Table 3**. In the multivariate analysis for OS, Cheng's PVTT type was found as an independent predictor of worse outcomes (95% CI: 1.311–2.253, HR = 1.719, *p < 0.001*), while salvage hepatectomy correlated with improved outcomes (95% CI: 0.273–0.777, HR = 0.460, *p = 0.004*). The multivariate analysis for PFS identified two independent risk factors: total bilirubin > 34.2 μmol/L (95% CI: 1.238–4.702, HR = 2.412, *p = 0.010*) and Cheng's PVTT type (95% CI: 1.308–2.091, HR = 1.654, *p < 0.001*). Salvage hepatectomy was an independent protective factor for PFS (95% CI: 0.341–0.845, HR = 0.537, *p = 0.007*). To further validate the survival benefit and eliminate immortal time bias, a time-dependent Cox analysis was conducted (**Supplementary Table S1**). The results confirmed that salvage hepatectomy remained an independent protective factor for OS (HR = 0.968, 95% CI: 0.939–0.998, *p*  = 0.036) and PFS (HR = 0.946, 95% CI: 0.905– 0.988, *p*  = 0.012), even after adjusting for the timing of surgery. Cheng's PVTT classification also persisted as a significant independent risk factor for both OS and PFS (*p*  < 0.001).

**Table 3:** Univariate and multivariate analysis of the total patients for OS and PFS.

Variables	PFS			OS		
	HR	95%CI	P value	HR	95%CI	P value
*Univariate*						
Age (≤60 / >60, y)	0.882	0.555-1.402	0.595	0.774	0.505-1.187	0.240
Sex (Female / Male)	1.330	0.645-2.740	0.440	1.228	0.622-2.421	0.554
AFP (≥400 / <400, μg/L)	1.333	0.901-1.970	0.150	1.145	0.803-1.630	0.455
AFP-L3 (<10 / ≥10)	1.481	1.008-2.175	***0.045***	1.324	0.928-1.887	0.121
PVK-II (≥400 / <400, μg/L)	1.667	1.099-2.529	***0.016***	1.453	1.003-2.105	***0.048***
TB ( ≥34.2/ <34.2, μmol/L)	2.901	1.540-5.464	***0.001***	3.180	1.697-5.958	***0.000***
ALB ( ≥40/ <40, g/L)	1.299	0.884-1.909	0.183	1.199	0.843-1.706	0.313
ALT (>40 / ≤40, IU/L)	1.074	0.732-1.576	0.714	0.967	0.681-1.375	0.854
AST (>40 / ≤40, IU/L)	1.615	1.096-2.380	***0.015***	1.616	1.134-2.304	***0.008***
HBV-DNA (<2000 / ≥2000, IU/mL)	1.037	0.690-1.560	0.860	1.094	0.755-1.583	0.636
ALBI grade (1/ 2/ 3)	1.540	1.051-2.258	***0.027***	1.328	0.938-1.880	0.110
TACE (No / Yes)	0.721	0.384-1.353	0.309	0.782	0.430-1.420	0.419
Livers cirrhosis (No / Yes)	1.015	0.691-1.492	0.938	1.166	0.819-1.661	0.395
HBsAg (Negative / Positive)	1.308	0.716-2.390	0.383	2.095	1.127-3.891	***0.019***
Cheng's PVTT type (I / II / III / IV)	1.689	1.298-2.198	***0.000***	1.555	1.226-1.973	***0.000***
Tumor number (Single / Multiple)	1.198	0.815-1.762	0.359	1.215	0.853-1.728	0.280
Tumor size (<10 / ≥10, cm)	1.053	0.710-1.563	0.796	1.085	0.756-1.556	0.657
Salvage hepatectomy (No / Yes)	0.410	0.247-0.683	***0.001***	0.487	0.314-0.755	***0.001***
*Multivariate*						
TB ( ≥34.2/ <34.2, μmol/L)				2.412	1.238-4.702	***0.010***
Salvage hepatectomy (No / Yes)	0.460	0.273-0.777	***0.004***	0.537	0.341-0.845	***0.007***
Cheng's PVTT type (I / II/ III/ IV)	1.719	1.311-2.253	***0.000***	1.654	1.308-2.091	***0.000***

OS, overall survival; PFS, progression free survival; HBsAg, hepatitis B surface antigen; TB, total bilirubin; ALBI, Albumin-Bilirubin Score; ALB, albumin; ALT, alanine transaminase; AST, aspartate aminotransferase; HBV-DNA, hepatitis B virus deoxyribonucleic acid; AFP, a-fetoprotein; AFP-L3, LCA-reactive alpha-fetoprotein isoform; TACE, transarterial chemoembolization; PVTT, portal vein tumor thrombus. P<0.05 was defined as statistical significance and indicated in bold italics.

The proportional hazards assumption of the Cox regression models was further evaluated using Schoenfeld residual tests (**Supplementary Table S2**). For the OS model, the global test did not show a significant violation of the PH assumption (global p = 0.114), although TB (p = 0.011) and ALBI grade (p = 0.030) showed potential deviations. For the PFS model, the global test also supported the overall PH assumption (global p = 0.145), although PIVKA-II (p = 0.012), TB (p = 0.037), AFP (p = 0.021), and ALBI grade (p = 0.042) showed possible deviations. Therefore, the hazard ratios of these individual variables should be interpreted with caution.

### AEs

The overall safety profile of the combined conversion therapy was manageable (**Table 4**). All reported AEs were recorded as treatment-related events occurring during the course of combined-modality therapy, and no treatment-related deaths were observed. The frequently reported AEs of any grade included liver function abnormalities (32.1%), fever (16.4%), hypertension (12.6%), skin rash (12.6%), and exhaustion (11.9%). Furthermore, AEs of grade ≥ 3 were observed in 19.5% of patients, with the most prevalent being gastrointestinal bleeding (5.0%), skin rash (5.0%), hypothyroidism (2.5%), proteinuria (2.5%), liver function abnormalities (2.5%), decreased appetite (1.3%), and tachypnea (0.6%). Among the 8 patients with gastrointestinal bleeding, 3 had received atezolizumab plus bevacizumab, whereas the remaining events occurred in patients treated with sorafenib (n = 2), lenvatinib (n = 2), and lenvatinib plus sintilimab (n = 1). Clinically, hypertension, proteinuria, hypothyroidism, and skin rash were more likely associated with systemic therapy, whereas fever and transient liver function abnormalities were more commonly observed after TACE; however, because of the retrospective design and overlapping toxicities, definitive attribution of each AE to a single treatment component was not feasible.

**Table 4:** Adverse events.

Symptoms	All (n=159)	
	Grade I-II	Grade III-IV
Any adverse event	132 (83.0%)	31 (19.5%)
Fatigue	19 (11.9%)	
Hypertension	20 (12.6%)	
Decreased appetite	14 (8.8%)	2 (1.3%)
Diarrhea	10 6.3%)	
Hypothyroidism	3 (1.9%)	4 (2.5%)
Proteinuria	4 (2.5%)	4 (2.5%)
Fever	26 (16.4%)	
Edema	4 (2.5%)	
Nausea	14 (8.8%)	
Vomiting	8 (5.0%)	
Hand-foot skin reaction	4 (2.5%)	
Skin rash	18 (11.3%)	8 (5.0%)
Abdominal pain	12 (7.5%)	
Abnormal liver function	49 (30.8%)	4 (2.5%)
Gastrointestinal hemorrhage		8 (5.0%)
Tachypnea		1 (0.6%)

### Salvage hepatectomy and perioperative conditions

In total, 40 patients underwent salvage hepatectomy after a median interval of 4.0 months (range, 2.3–7.8) from the start of conversion therapy. **Table 5** indicates surgical details and pathological findings. Multiple therapy protocols were administered before surgery, predominantly the combination of Lenvatinib with a PD-1 inhibitor and TACE, administered to 24 (60.0%) patients, followed by co-treatment with Lenvatinib and TACE, administered to 10 (25.0%) patients. Alternative regimens, including Apatinib, Donafenib, and Sorafenib, were employed in a few cases. The median surgical duration was 180 minutes (3.0 hours), and the median hepatic portal occlusion duration was 22.5 minutes (range, 15.3–31.0 minutes). Moreover, 17 patients (42.5%) experienced intraoperative blood loss > 400 mL, and 13 (32.5%) required blood transfusions. Pathological investigation indicated the presence of microvascular invasion (MVI) in 21 patients (52.5%), a tumor capsule was detected in 20 patients (50.0%), and satellite lesions were discovered in 25 individuals (62.5%). A pCR was attained in 11 patients (27.5%), while an MPR was found in 13 patients (32.5%). No significant postoperative problems were noted.

**Table 5:** Perioperative outcomes of conversion group.

Variables	Patients
	n=40	
Treatment regimens	
Apatinib	2
Apatinib + anti-PD-1 antibodies + TACE	1
Donafenib + anti-PD-1 antibodies + TACE	1
Lenvatinib + TACE	10
Lenvatinib + PD-1 + TACE	24
Sorafenib + tislelizumab + TACE	1
Lenvatinib + tislelizumab	1
Blood loss, ml	
<400	23 (57.5%)
≥400	17 (42.5%)
Blood transfusion	
Yes	13 (32.5%)
No	27 (67.5%)
Operative time, h, median (Q1, Q3)	3.0 (3.0, 3.5)
Surgical margin, cm, median (Q1, Q3)	0.5 (0.2, 1.0)
Hepatic portal occlusion time, min, median (Q1, Q3)	22.5 (15.3, 31.0)
Pathological reaction	
PCR	11 (27.5%)
MPR	13 (32.5%)
Non-MPR	16 (40.0%)
MVI	
M0	19 (47.5%)
M1	10 (25.0%)
M2	11 (27.5%)
Tumor capsule	
Yes	20 (50.0%)
No	20 (50.0%)
Satellite lesions	
Yes	25 (62.5%)
No	15 (37.5%)

TACE, transcatheter arterial chemoembolization; PD-1, PD-1 antibody; PCR, pathological complete response; MPR, major pathological response; MVI, microvascular invasion.

Of the 40 patients who underwent surgery, 25 (62.5%) experienced tumor recurrence during follow-up. The most common pattern was intrahepatic recurrence (n=17, 68.0%), followed by extrahepatic metastasis (n=5, 20.0%) and combined recurrence (n=3, 12.0%). The extrahepatic sites included the lungs, bones, and lymph nodes.

### Predictors and survival outcomes of salvage hepatectomy

**Figure 2:**
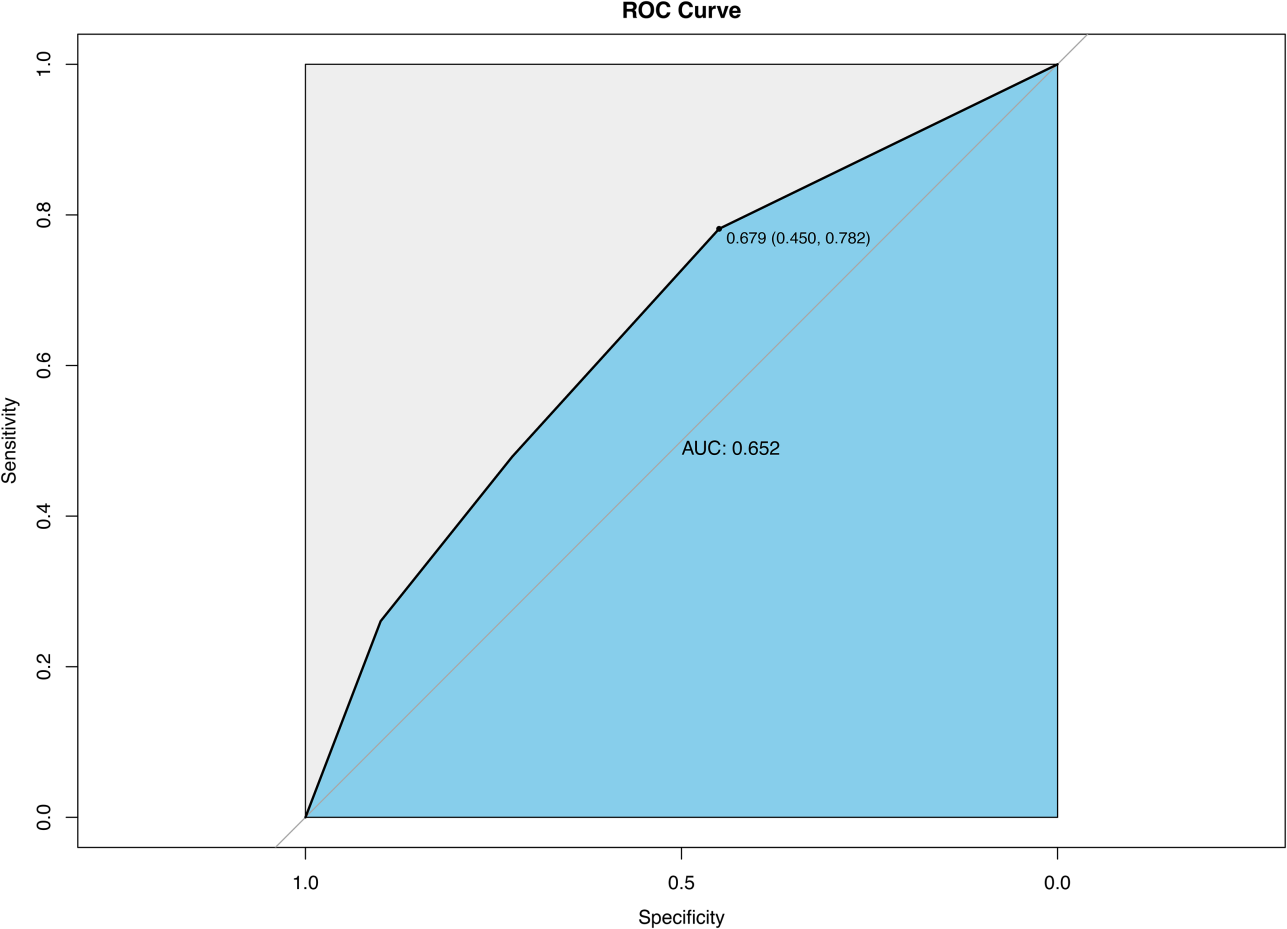
ROC curve for predicting successful conversion to salvage hepatectomy. ROC, Receiver operating characteristic. The area under the curve (AUC) is 0.652 (95% CI: 0.558–0.746).

The potential factors related to successful conversion to hepatectomy in patients with initially uHCC were assessed (**Table 6**). Multivariate logistic regression analysis revealed that the baseline aspartate aminotransferase (AST) levels (95% CI: 0.214– 0.958, OR = 0.453, *p = 0.038*) and tumor number (95% CI: 0.182–0.888, OR = 0.402, *p = 0.024*) were independent predictors of successful conversion. To further evaluate the model’s performance, ROC analysis was conducted. The combined model, incorporating baseline AST levels and tumor number, demonstrated a significant discriminative ability for successful conversion, with an AUC of 0.652 (95% CI: 0.558–0.746, **Figure 2**).

**Table 6:** Logistic regression analysis of the conversion group.

Variables	B	SE	Wald	df	Odds ratio	95%CI	P value
AST (>40 / ≤40, IU/L)	-0.792	0.382	4.297	1	0.453	0.214-0.958	***0.038***
Tumor number (Single / Multiple)	-0.911	0.404	5.074	1	0.402	0.182-0.888	***0.024***

AST, aspartate aminotransferase. P<0.05 was defined as statistical significance and indicated in bold italics

**Figure 3:**
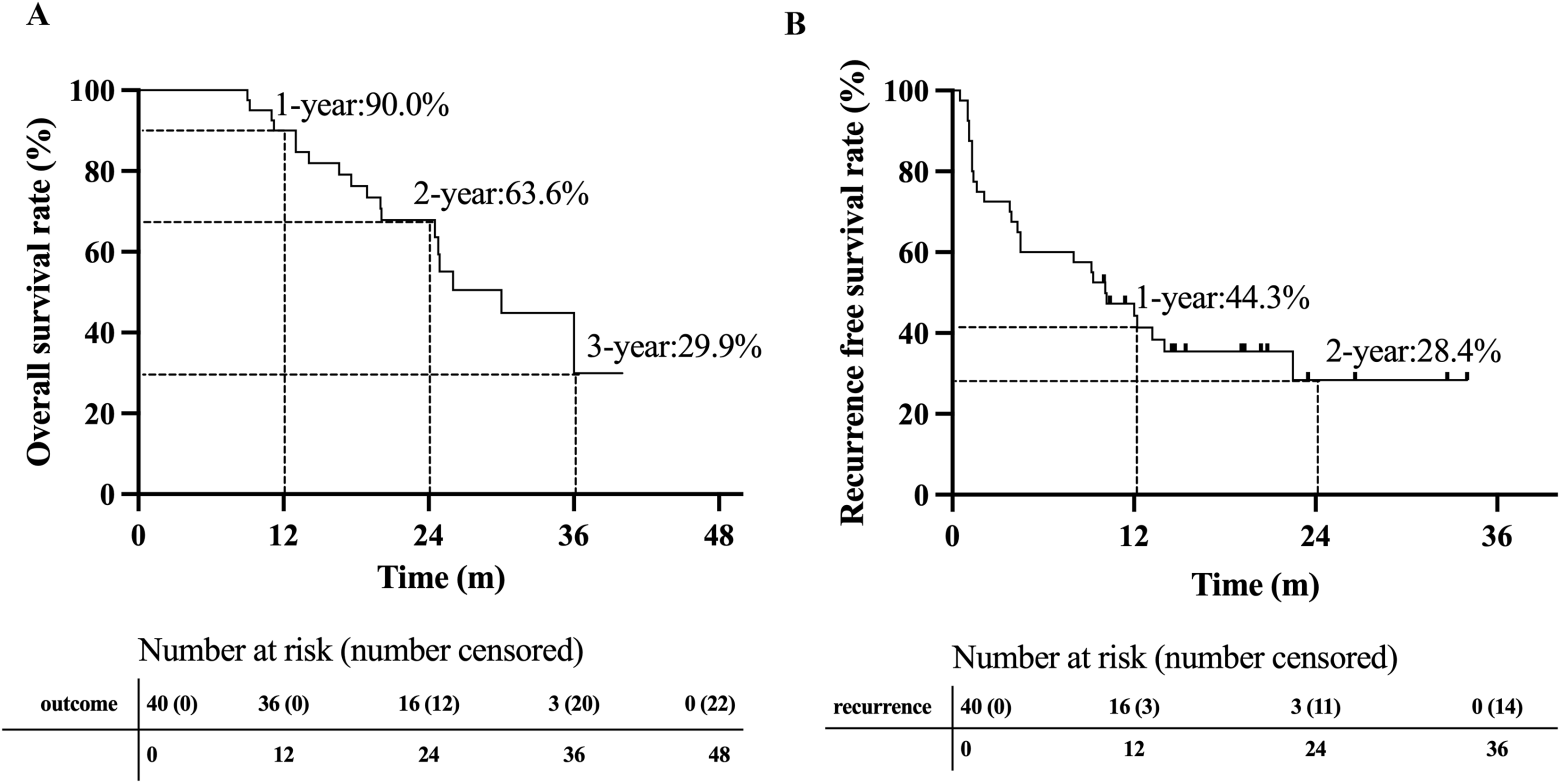
Kaplan-Meier curves in the conversion therapy group for uHCC patients with Portal Vein Tumor Thrombus. **(A)** Overall survival (OS). **(B)** Recurrence free survival (RFS). HCC, hepatocellular carcinoma.

For patients who underwent conversion therapy and subsequent salvage hepatectomy, the median OS and RFS were 30.0 and 10.1 months, respectively. The respective 1-, 2-, and 3-year cumulative OS rates were determined to be 90.0%, 63.6%, and 29.9%. The corresponding 1- and 2-year RFS rates were 44.3% and 28.4%, respectively (**Figures 3A, B**).

### Survival outcomes based on pathological response

**Figure 4:**
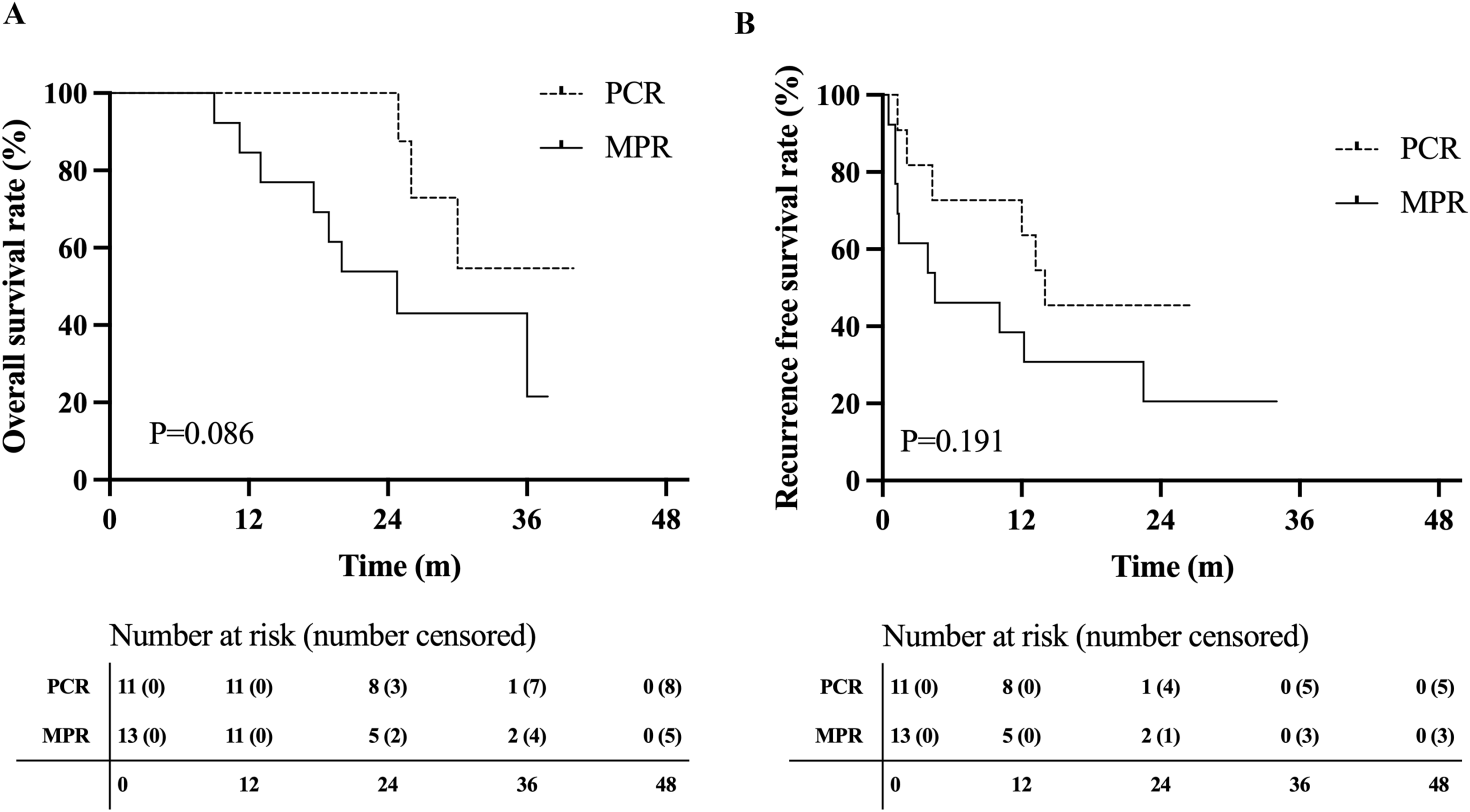
uHCC patients Kaplan-Meier curves in the conversion therapy group stratified by pathological response for OS **(A)** and RFS **(B)**. **(A)** Overall survival (OS). **(B)** Recurrence free survival (RFS). HCC, hepatocellular carcinoma.

Among patients in the conversion therapy group, 11 (27.5%) achieved a pCR and 13 (32.5%) achieved an MPR. Patients with pCR showed numerically more favorable survival outcomes than those with MPR, and the median OS was not reached in the pCR group. The respective 1-, 2-, and 3-year cumulative OS rates were determined to be 100.0%, 87.5%, and 54.7%. Whereas for MPR patients, the median OS was 24.8 months, with corresponding 1-, 2-, and 3-year OS rates of 84.6%, 43.1%, and 21.5%. Moreover, the RFS was numerically prolonged in the pCR group, with a median RFS of 14.0 months, in contrast to 4.5 months in the MPR group. The pCR group's 1- and 2-year RFS rates were 63.6% and 45.5%, respectively, compared to 30.8% and 20.5% for the MPR group. However, no statistically significant difference was observed in OS or RFS between the pCR and MPR groups (**Figures 4A, B**, all *p > 0.05*), which might be because of the limited sample size of the surgical cohort. Therefore, these findings should be interpreted cautiously and considered exploratory.

## Discussion

This retrospective study evaluated 159 uHCC patients with PVTT, who received conversion therapy combining systemic and locoregional approaches, primarily including TACE as the locoregional modality. This comprehensive strategy indicated a promising ORR (42.1%) and DCR (62.3%). Furthermore, this method achieved successful down-staging in 25.2% of patients via conversion therapies, making them suitable for curative-intent salvage hepatectomy. The multivariate study validated that salvage hepatectomy was an independent protective factor for both PFS and OS, while a more advanced baseline Cheng's classification of PVTT was a significant indicator of an unfavorable outcome.

The findings of this investigation are consistent with the paradigm shift in the management of advanced HCC. Historically, HCC patients with PVTT have a substandard prognosis, with only a few months of median survival (14, 15). However, its treatment strategies have been revolutionized because of targeted therapies and ICIs, especially following the pivotal IMbrave150 trial, which demonstrated the superiority of atezolizumab combined with bevacizumab as the first-line standard of care (4). The many combinations of targeted therapies and immunotherapies employed in this cohort illustrate the therapeutic application of this novel paradigm. The observed ORR of 42.1% is comparable to, or surpasses, rates documented in key studies such as IMbrave150 (around 30%). The improved efficacy may be due to the substantial percentage of patients (91.8%) who received concomitant TACE. However, without a systemic-therapy-alone control arm, the exact additive or synergistic benefit of TACE cannot be definitively quantified in this study.

TACE represents the established therapeutic approach for patients with intermediate-stage HCC, demonstrating high ORR and a favorable safety profile (16). TACE's therapeutic mechanism is primarily attributed to its ability to induce ischemic necrosis by increasing tumor hypoxia. However, TACE-induced hypoxia might paradoxically facilitate tumor progression by upregulating hypoxia-related proteins, which subsequently enhance pro-angiogenic molecules such as fibroblast growth factor (FGF) and vascular endothelial growth factor (VEGF) (17). This limitation promotes the application of combination therapies. For example, the combination of TACE with Lenvatinib, a multi-kinase inhibitor, can mitigate this pro-angiogenic surge by directly blocking VEGF receptor activation, resulting in synergistic anticancer benefits (18). TACE may stimulate the immune system by generating significant tumor necrosis and the release of tumor-associated antigens, thereby increasing systemic anti-tumor immune responses through an abscopal effect when used in conjunction with immunotherapies (19, 20).

This research study primarily investigated the substantial influence of effective conversion therapy on prolonged outcomes. In the whole cohort, 40 patients (25.2%) received salvage hepatectomy, resulting in a significant median OS of 30.0 months, significantly above that of the non-surgical group. These findings highlight that, for uHCC patients with PVTT, conversion therapy is an effective and critical strategy for enhancing long-term survival. Furthermore, the multivariate analysis further corroborated that salvage hepatectomy serves as a significant independent protective factor for OS (HR = 0.460, *p = 0.004*) and PFS (HR = 0.537, *p = 0.007*). This conclusion is consistent with a previous literature suggesting that converting uHCC to a resectable state is the most effective for achieving long-term survival (21–23). Importantly, we acknowledge that retrospective comparisons involving salvage hepatectomy are susceptible to immortal time bias, because patients must survive and maintain adequate response long enough to become eligible for surgery. To address this issue, we further performed a time-dependent Cox regression analysis treating salvage hepatectomy as a time-dependent variable. It is important to contextualize the substantial difference in hazard ratio (HR) magnitudes between the standard Cox (HR = 0.460 for OS) and the time-dependent Cox (HR = 0.968 for OS). In the standard model, surgery is treated as a fixed baseline variable, which inherently credits the surgical group with the guaranteed survival period needed to reach surgery (immortal time bias), thereby artificially inflating its apparent protective effect. In contrast, the time-dependent model corrects for this by shifting patients to the surgical group only at the actual time of the operation. Consequently, the time-dependent HR (0.968) represents a more conservative, unconfounded measure of the actual risk reduction conferred by the surgery itself. The survival benefit of salvage hepatectomy remained significant for both OS and PFS in this additional analysis, indicating that the favorable outcomes observed in the surgical group were not merely the result of immortal time bias. This strengthens the validity of our conclusion that successful conversion to salvage hepatectomy confers a real survival advantage in selected patients with uHCC and PVTT. Moreover, it was found that reduced baseline AST levels and a single tumor are independent predictors of successful conversion. In addition, increased AST levels indicate active hepatocyte necrosis and hepatic inflammation, especially in individuals with liver cirrhosis. The persistent inflammatory condition, observed in cirrhosis and chronic active hepatitis, is a recognized risk factor for intrahepatic recurrence of HCC (24). Thus, by indicating the degree of hepatic inflammation, AST can markedly predict long-term survival outcomes (25). A reduced AST level may indicate improved liver functional reserve and diminished tumor aggressiveness, whereas a single tumor is intrinsically more susceptible to locoregional therapy. These parameters may serve as significant clinical indicators for identifying patients most likely to benefit from a conversion strategy.

The optimal duration of conversion therapy before surgery remains controversial. While a median interval of 4 months in our study provided a window for salvage hepatectomy, the median RFS of 10.1 months underscores the persistent high risk of recurrence in PVTT patients. This suboptimal RFS suggests that even with a significant imaging response, microscopic residual disease or circulating tumor cells may lead to early recurrence. Future studies should explore whether extending the preoperative treatment duration or intensifying postoperative adjuvant therapy could further prolong RFS in this population.

Here, the pathological response of the surgical subgroup revealed that patients who achieved a pCR had the most favorable survival outcomes, did not reach a median OS, and had a 3-year OS rate of 54.7%. The lack of statistical significance in survival differences between the pCR and MPR groups might be attributed to the small sample size; however, there was a marked difference in the survival curves. This pattern corresponds with studies on other solid tumors, including breast and lung cancer, where pCR is a recognized surrogate endpoint for long-term therapeutic advantage (26, 27). These findings suggest that deeper pathological response may be clinically relevant; however, given the lack of statistical significance and the limited sample size, the prognostic value of pCR should be considered hypothesis-generating and requires validation in larger studies.

The safety profile of the combination therapy was manageable; however, there are significant toxicities. The predominant serious AEs were gastrointestinal bleeding (5.0%) and skin rash (5.0%). Gastrointestinal bleeding is substantially dangerous in HCC patients, especially in those with portal hypertension or those undergoing treatment with anti-angiogenic drugs such as bevacizumab or tyrosine kinase inhibitors, which may compromise vascular integrity (28). Moreover, severe skin reactions are recognized as toxicities of both TKIs and ICIs (29, 30). The lack of treatment-related deaths suggests that, with proactive monitoring and management by an experienced MDT, these strategies can be administered safely to appropriately selected patients with a favorable PS and liver function.

Certain limitations are associated with the current study. (1) This single-center study has inherent selection and information biases. (2) The systemic treatment regimens used in this cohort were heterogeneous, encompassing various TKIs and PD-1 inhibitors. This reflected the real-world clinical setting and personalized MDT decisions, but it limited our ability to evaluate the efficacy of any single drug. However, the consistent application of the combined modality across the cohort suggests that the synergistic strategy is the primary driver of successful conversion, rather than a specific agent. Future prospective trials with standardized drug regimens are needed to refine the optimal combination. (3) The number of patients who underwent salvage surgery was small and may have restricted the statistical power for subgroup analyses, including the comparison of pCR and MPR outcomes. (4) Confounding by treatment response is an important limitation of this study. Patients who were successfully converted to surgery likely represented a subgroup with more favorable tumor biology and greater treatment sensitivity. As salvage hepatectomy was a post-baseline, response-dependent intervention, its apparent survival benefit cannot be fully separated from the prognostic effect of treatment response itself. Although we additionally performed multivariable adjustment and a time-dependent Cox analysis to reduce immortal time bias related to salvage hepatectomy, residual confounding and selection bias could not be completely eliminated due to the retrospective design. (5) Crucially, the absence of a matched control group receiving systemic therapy alone prevents us from concluding that the TACE-based combination therapy is superior to systemic therapy alone. Therefore, our findings reflect the overall feasibility of this combined strategy rather than its superiority over monotherapy. (6) The follow-up time is still developing, and extended data are required to validate these findings.

## Conclusion

In summary, this study indicated that a comprehensive conversion therapy, integrating TACE and modern systemic therapies, is safe and effective for uHCC patients with PVTT. This strategy provides high tumor response rates and a cure for a subset of patients, therefore markedly improving their long-term survival. Furthermore, it was found that baseline Cheng’s PVTT categorization serves as a vital prognostic indicator; however, successful salvage hepatectomy is the most significant predictor for a favorable outcome. Future studies (specifically, prospective, multi-center trials) should optimize treatment combinations to improve conversion rates and the extent of pathological response.

## Data Availability

The raw data supporting the conclusions of this article will be made available by the authors, without undue reservation.

## Supplementary material

Supplementary Table 1 Time-dependent analysis of the total patients for OS and PFS.

Supplementary Table 2 Proportional hazards assumption for both the OS and PFS Cox regression models.
