# Supplementary material for: Efficacy of combined modality conversion therapy for HCC with portal vein tumor thrombus: a retrospective cohort study
Source: Front Immunol. 2026 Jun 18;17:1749090. doi: 10.3389/fimmu.2026.1749090 (PMC13323150; doi:10.3389/fimmu.2026.1749090)
Supplement: Supplementary Table 1 — Time-dependent analysis of the total patients for OS and PFS. [file Table1.docx]

| **Table S1. Time-dependent analysis of the total patients for OS and PFS** | | | | | | | |
| --- | --- | --- | --- | --- | --- | --- | --- |
|  | OS | | |  | PFS | | |
| Variables | HR | 95%CI | P value |  | HR | 95%CI | P value |
|  |  |  |  |  |  |  |  |
| Age (≤60 / >60, y) | 0.996 | 0.612-1.622 | 0.988 |  | 0.813 | 0.517-1.276 | 0.368 |
| Sex (Female / Male) | 1.204 | 0.563-2.574 | 0.632 |  | 1.297 | 0.632-2.663 | 0.478 |
| AFP-L3 (<10 / ≥10) | 1.367 | 0.904-2.067 | 0.138 |  | 1.404 | 0.966-2.041 | 0.075 |
| PVK-II (≥400 / <400, μg/L) | 1.394 | 0.879-2.212 | 0.158 |  | 1.280 | 0.850-1.927 | 0.238 |
| ﻿TB (≥34.2 / <34.2, μmol/L) | 1.947 | 0.944-4.017 | 0.071 |  | 2.907 | 1.412-5.985 | ***0.004*** |
| ALBI grade (1 / 2 / 3) | 1.063 | 0.700-1.614 | 0.776 |  | 0.862 | 0.590-1.260 | 0.443 |
| Livers cirrhosis (No / Yes) | 0.717 | 0.472-1.090 | 0.119 |  | 0.876 | 0.594-1.292 | 0.504 |
| Cheng's PVTT type (I / II / III / IV) | 1.733 | 1.315-2.285 | ***<0.001*** |  | 1.684 | 1.315-2.156 | ***<0.001*** |
| Tumor number (Single / Multiple） | 1.056 | 0.686-1.624 | 0.806 |  | 1.036 | 0.707-1.520 | 0.856 |
| Tumor size (<10 / ≥10, cm) | 1.158 | 0.753-1.783 | 0.504 |  | 1.257 | 0.852-1.856 | 0.250 |
| Salvage hepatectomy (No / Yes) | 0.968 | 0.939-0.998 | ***0.036*** |  | 0.946 | 0.905-0.988 | ***0.012*** |
| OS, overall survival; PFS, progression free survival; TB, total bilirubin; ALBI, Albumin-Bilirubin Score; AFP-L3, LCA-reactive alpha-fetoprotein isoform; PVTT,﻿ portal vein tumor thrombus. | | | | | | | |
| ﻿P<0.05 was defined as statistical significance and indicated in bold italics. | | | | | | | |
