# Supplementary material for: Efficacy of combined modality conversion therapy for HCC with portal vein tumor thrombus: a retrospective cohort study
Source: Front Immunol. 2026 Jun 18;17:1749090. doi: 10.3389/fimmu.2026.1749090 (PMC13323150; doi:10.3389/fimmu.2026.1749090)
Supplement: Supplementary Table 2 — Proportional hazards assumption for both the OS and PFS Cox regression models. [file Table2.docx]

| **Table S2. Proportional hazards assumption for both the OS and PFS Cox regression models** | | | | | | |
| --- | --- | --- | --- | --- | --- | --- |
|  |  | OS | |  | PFS | |
|  |  | Chisq | P |  | Chisq | P |
| Age |  | 0.555 | 0.456 |  | 0.103 | 0.748 |
| Sex |  | 1.114 | 0.291 |  | 1.560 | 0.211 |
| AFP |  | 1.957 | 0.162 |  | 5.370 | ***0.021*** |
| AFP-L3 |  | 0.646 | 0.422 |  | 0.277 | 0.599 |
| PVK-II |  | 2.821 | 0.093 |  | 6.350 | ***0.012*** |
| ﻿TB |  | 6.404 | ***0.011*** |  | 4.360 | ***0.037*** |
| ALB |  | 1.278 | 0.258 |  | 0.789 | 0.375 |
| ALT |  | 0.177 | 0.674 |  | 0.973 | 0.324 |
| AST |  | 0.694 | 0.405 |  | 0.296 | 0.586 |
| HBV-DNA |  | 2.016 | 0.156 |  | 0.202 | 0.653 |
| ALBI grade |  | 4.698 | ***0.030*** |  | 4.140 | ***0.042*** |
| TACE |  | 1.594 | 0.207 |  | 0.349 | 0.555 |
| Livers cirrhosis | | 2.844 | 0.092 |  | 0.573 | 0.449 |
| HBsAg |  | 3.241 | 0.072 |  | 0.272 | 0.602 |
| Cheng's PVTT type | | 3.079 | 0.079 |  | 0.743 | 0.389 |
| Tumor number | | 2.049 | 0.152 |  | <0.001 | 1.000 |
| Tumor size |  | 2.191 | 0.139 |  | 0.211 | 0.646 |
| Salvage hepatectomy | | 3.652 | 0.056 |  | 0.849 | 0.357 |
| GLOBAL |  | 5.408 | 0.114 |  | 24.300 | 0.145 |
| OS, overall survival; PFS, progression free survival; HBsAg, hepatitis B surface antigen; TB, total bilirubin; ALBI, Albumin-Bilirubin Score; ALB, albumin; ALT, alanine transaminase; AST, aspartate aminotransferase; HBV-DNA, hepatitis B virus deoxyribonucleic acid; AFP, a-fetoprotein; AFP-L3, LCA-reactive alpha-fetoprotein isoform; TACE, transarterial chemoembolization; PVTT,﻿ portal vein tumor thrombus. | | | | | | |
| ﻿P<0.05 was defined as statistical significance and indicated in bold italics. | | | | | |  |
